# Behavior and Performance Analysis of Fire Protection Materials Applied to Steel Structures According to Exposed Temperatures

**DOI:** 10.3390/ma18061285

**Published:** 2025-03-14

**Authors:** Hyun Kang, Oh-Sang Kweon

**Affiliations:** Korea Institute of Civil Engineering & Building Technology (KICT), 182-64 Mado-ro, Mado-myeon, Hwaseong-si 18544, Gyeonggi-do, Republic of Korea; kanghty@kict.re.kr

**Keywords:** steel frame structure, protection of steel, spray-on fireproofing material, intumescent paint, fire damage assessment

## Abstract

Spray-on fireproofing materials (SFRMs) and intumescent paints are commonly used to enhance the fire resistance of steel structures. Despite extensive studies on the performance of SFRMs and intumescent paints, there remains a significant lack of research on the material properties of certified products used in real-world applications, especially according to exposed temperatures. In this study, heating experiments were conducted to investigate the material properties of two widely used certified materials in Korea, focusing on their application thickness and exposure temperature. The experiments set target temperatures ranging from room temperature to 900 °C in increments of 100 °C. Additionally, various material properties, such as changes in shape and the insulation performance of the SFRM and intumescent paint, were observed at each temperature. Notably, the moisture content and discoloration depth of the SFRM displayed a consistent trend under different exposure temperatures, a material property that has not been previously reported. Furthermore, the insulation performance of the two materials differed by approximately 17% to 25% compared to an uncoated specimen exposed to the same temperature. The findings on the properties of SFRMs and intumescent paint relative to the exposure temperature presented in this study can serve as valuable data for selecting materials to improve fire resistance performance across various construction sites in the future. Additionally, they can act as key reference data in the diagnostic evaluation process for assessing the impact of fire on steel structures.

## 1. Introduction

Fires in buildings cause numerous casualties, as demonstrated by past tragedies. These include the Grenfell Tower fire in 2017 and the 2022 Bronx apartment fire, which helped raise global awareness about building fires. After the Great Fire of London in 1666, the earliest regulations on building fires were promoted mainly by insurance companies who were more interested in protecting property. Later, related regulations were established with a focus on preserving life. Numerous researchers have conducted experimental and theoretical studies, and now, each country has fire resistance regulations for buildings. Each regulation requires different fire resistance performance standards for structural members depending on the use and scale of the building. If the required performance standards are not met, various strengthening methods are applied to secure fire resistance performance.

Reinforced concrete structures generally have relatively low thermal conductivity and temperature-dependent strain rates. They are often used as fire-resistant structures without additional reinforcement because the concrete (with its high heat capacity) features high thermal conductivity and temperature strain, which helps protect the rebar with low thermal conductivity as long as there is sufficient cover thickness [1,2]. On the other hand, in steel frame structures, all the frames except the floor slab are made of steel. Therefore, fireproof reinforcement is necessary to ensure adequate fire resistance performance as mandated by regulations for fire protection [3,4,5]. Common fire protection materials include concrete covers, fireproof boards, spray-on fireproofing materials (SFRMs), and intumescent paints. Among them, spray-on fireproofing and intumescent paints are used relatively frequently due to their potential in regards to plane application and how well they facilitate the ease of construction [6,7].

In previous studies, various structural members have been reinforced with spray-on fireproofing and intumescent paints, and performance tests and analytical research have been conducted using standard fire curves [8,9,10]. Most of them aimed to assess the fire resistance performance of structural members and improve their performance. However, there is a lack of research on the fundamental behavior and performance of fire protection materials. However, some studies have provided various information on fire protection materials by analyzing the characteristics of the material composition of SFRMs and intumescent paints.

Kodur and Shakya [11] explained that SFRMs are widely used due to their cost-effectiveness, ease of application, and light weight. However, the material properties of SFRMs needed to perform analytical behavioral assessments of many structural members to which they are applied are not readily available. To address this issue, the researchers highlighted the influence of temperature on the thermal properties of different types of commercially available SFRMs. These results minimize the need for relatively costly and time-consuming experimental studies and allow for analytical studies, an alternative research method. In a similar study, Kang et al. [3] analyzed the behavior of structural members exposed to standard fires using material properties obtained through separate tests on fire protection materials commonly used in Korea. Li et al. [12] developed a fire-resistant coating material using natural brucite as a magnesium source, leveraging the high strength and high-temperature resistance of Magnesium Phosphate Cement (MPC) and its excellent bonding performance with steel members. As a result, the material they developed demonstrated improved properties, including increased strength and reduced thermal conductivity compared to existing MPC, as confirmed through various experiments. However, insulation performance testing was conducted solely with an alcohol lamp, which did not consider a range of exposure temperatures. Caetano et al. [13] presented the material properties of gypsum-based SFRMs with and without silica using both destructive and non-destructive tests. However, testing was limited to room temperature conditions, making it impossible to assess the characteristics of fire-damaged materials. Sun et al. [14] developed an alkali-activated cement-based fire protection material to address the limitations of conventional cement-based options. Although the researchers conducted various performance evaluations, the insulation performance test was also limited to an alcohol lamp exposure. In other words, like Li et al. [12], varying exposure temperatures were not considered in the study.

In recent years, intumescent paints have gained popularity despite their relatively high cost compared to SFRMs, as they offer an aesthetic appeal similar to that of general paint finishes. Additionally, they provide good insulation in the event of a fire. Consequently, numerous studies have been conducted on intumescent paints, including research on adjusting the ratios of various components to enhance the performance of existing paints [15], using nanotubes [16,17], and developing new materials using geopolymers that match the expansion properties of intumescent paints [18]. Other studies have focused on the performance of steel plates and beams with and without fireproof reinforcement [19,20]. Zielecka et al. [21] explored the expansion mechanism of silicone resin-based intumescent paint due to chemical reactions when heated and the resulting improvements in fire resistance performance. Gravit et al. [22] conducted performance tests on three materials (fireproof board, SFRM, and intumescent paint) under jet fire conditions encountered in industrial settings rather than standard fire conditions to compare the effectiveness of various reinforcement materials. Häßler et al. [23] presented a method for assessing the durability of intumescent coatings and how they can be improved. Intumescent paints are applied to general buildings as well as special-purpose structures such as large industrial complexes. Depending on the region where each structure is located and various environmental factors, physical damage that can be identified by visual inspection, such as coating failures, and chemical damage that cannot be identified by visual inspection may occur. Häßler et al. [23] noted that surface cracks, erosion, lifting, and reduced expansion effects in fires may occur in intumescent coatings due to the aging and weathering of materials. Therefore, they proposed a durability evaluation method based on EAD 350402-00-1106 [24] for long-term maintenance of intumescent paints.

As discussed above, most existing studies on SFRMs and intumescent paints have focused on improving fire protection performance, with some research conducted for maintenance purposes. This study aims to analyze the behavior and performance of spray-on fireproofing materials and intumescent paints at various exposure temperatures, which have not been previously studied. In addition to material heating experiments, various performance tests were conducted on cooled specimens to analyze their behavior and properties. This study was conducted as part of a research project to develop diagnostic evaluation procedures for fire-damaged steel structures, and all experimental results will be used as essential data in future diagnostic evaluation procedures.

## 2. Material Heating Experiment

### 2.1. Fire Protection Materials (Spray-On Fireproofing and Intumescent Paint)

Spray-on fireproofing materials and intumescent paints, which are commonly applied to improve the fire resistance performance of steel frame structures, secure the fire resistance performance of steel members. This is achieved through a mechanism that delays the time for the temperature of the flame to reach the member through the insulation performance of each material in the event of a fire. SFRMs exhibit insulation effects due to the low thermal conductivity of the material itself without any physical or chemical changes. Meanwhile, intumescent paints form an air insulation layer on the surface of the member as the ammonium polyphosphate begins to decompose [21], causing it to expand at high temperatures (200–300 °C) in the event of a fire, thereby providing insulation effects and fire resistance performance. These two fire protection materials are applied to members through a separate process at the construction site, and in the case of the SFRM, the construction time is relatively short and the cost of the process is low. In the case of intumescent paint, a layer of paint of a specific thickness is applied according to the required fire resistance performance time. This paint is then cured and re-applied to achieve the appropriate coating thickness. This process lengthens the construction time and increases the cost. In addition, particular attention should be paid to the maintenance of each fire protection material evenly applied to the surface of the member. This is because defects such as surface cracking, lifting, and falling off can lead to a rapid deterioration in the performance of the member due to a decrease in the insulation effect of the relevant area in the event of a fire. This was demonstrated by the 11 September 2001 terrorist attacks in the United States, where the localized failure of sprayed fire-resistive material applied to the truss structure of the floor slab was noted as one of the various causes that led to the collapse of the World Trade Center [25]. In addition, the poor application and removal of reinforcing materials in some areas during the construction and maintenance process, along with the impact of the aircraft crashing into the buildings, have been suggested as causes of the failure of the sprayed fire-resistive material [25].

### 2.2. Heating Experiments

**Figure 1:**
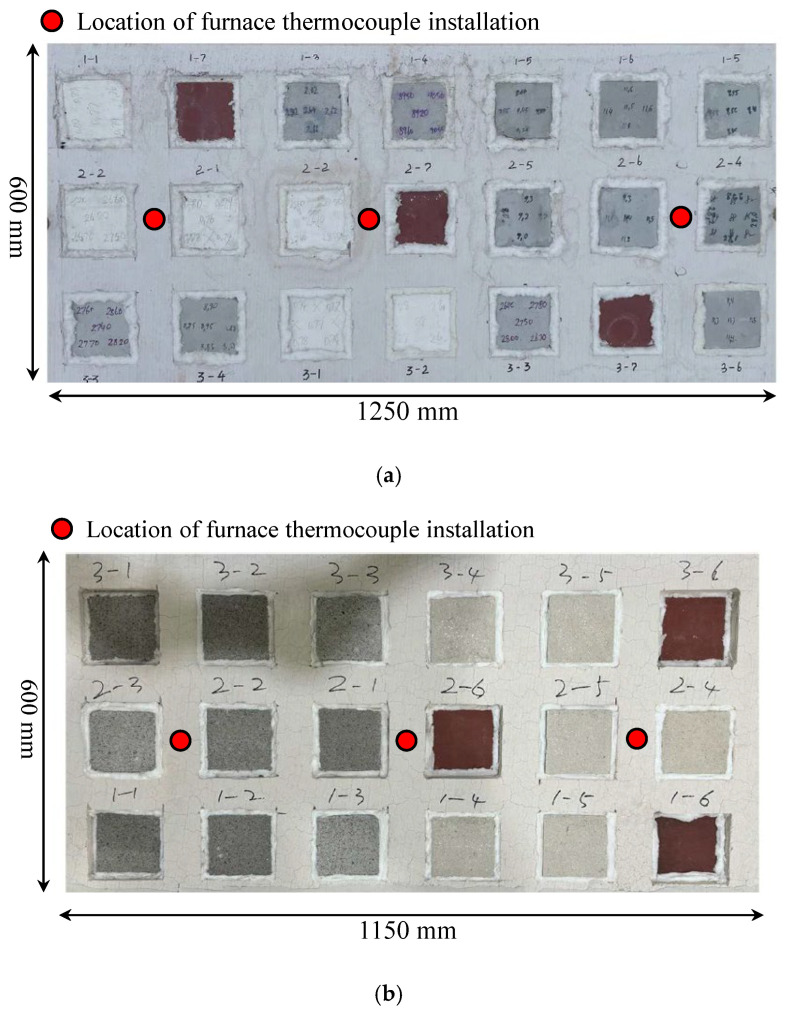
Specimen panel (fire-exposed area). (**a**) Intumescent paint specimen; (**b**) spray-on fireproofing specimen.

As part of a research project to develop diagnostic evaluation procedures for fire-damaged steel structures, this study analyzed the behavior and performance of SFRMs and intumescent paints frequently applied to steel frame structures under different temperatures. The behavior and performance data of fire protection materials exposed to different temperatures can be used as a database for diagnostic evaluation procedures to be developed in the future. They can also be used as a means to assess the temperature in the event of a fire according to the characteristics of each material. Table 1 shows the variables for material heating experiments. The tests on the SFRM and intumescent paints were conducted in separate furnaces due to the influence of damage in using the same furnace. Each specimen was prepared by applying SFRMs and intumescent paints to steel plates of the same size, as shown in Table 1. A K-type thermocouple with a diameter of 0.5 mm was installed at the center of each specimen’s steel plate. To shorten the duration of the experiments, insulation panels, as shown in Figure 1, were used to arrange all specimens targeting the same temperature on a single panel. This setup allowed for the implementation of one heating experiment for each target temperature. Three specimens with identical reinforcement variables were prepared and placed for each target temperature. Consequently, as illustrated in Figure 1, the SFRM specimens included six reinforcement variables, resulting in 18 specimens, while the intumescent paint specimens comprised seven reinforcement variables, resulting in a total of 21 specimens. ‘Data ID’ in Table 1 indicates each specimen for the respective variable, which can be referenced in the data for the results. Furthermore, the reinforcement thickness for each material was based on the thickness of the products certified in Korea to meet the required fire resistance performance within a timeframe of 1 to 3 h.

**Figure 2:**
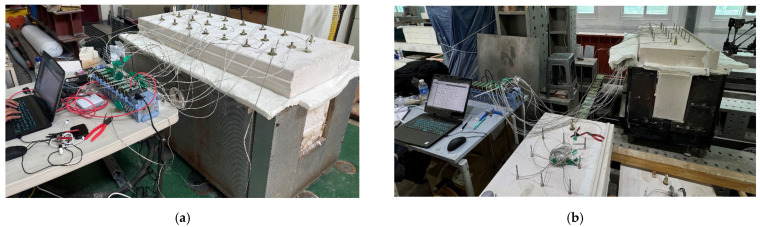
Experimental setup. (**a**) Intumescent paint; (**b**) spray-on fireproofing.

The heating experiment was conducted by placing the panel from Figure 1 on top of the furnace, as depicted in Figure 2, using a one-sided heating method. This design allowed for the measurement of the temperature reaching the steel plate from the external flame temperature while analyzing the properties of the reinforcement material under conditions replicating those of actual structures. The furnace had internal dimensions of 60 mm × 60 mm × 1350 mm and could control temperatures from 20 to 1100 °C. It had a heating capacity of 9.6 kW and operated on an alternating current power source with a frequency of 50 to 60 Hz.

The heating experiments were performed on steel plate specimens with high thermal conductivity. Therefore, the heating rate was set to 10 °C/min before conducting tests at temperatures of 100 °C and 200 °C on the intumescent paint panel, the first test specimen. However, the heating rate was adjusted to 5 °C/min in subsequent experiments after confirming that the temperature of the uncoated specimen (‘IP_N’ in Table 1) did not reach the target temperature due to the one-sided heating conditions.

**Figure 3:**
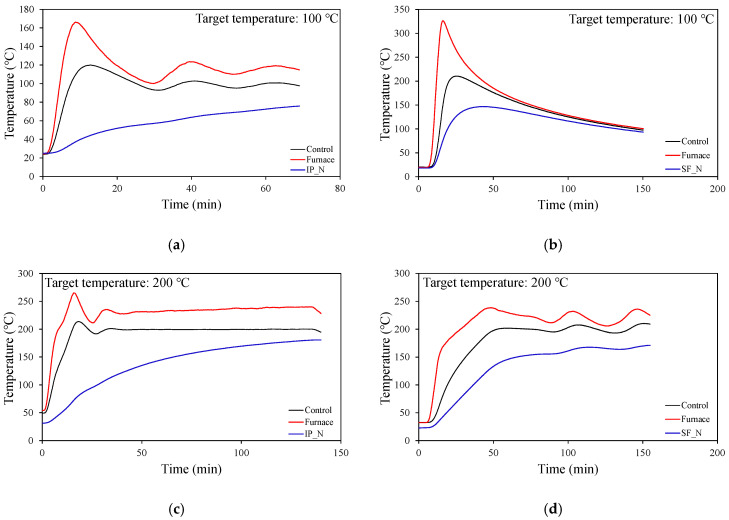

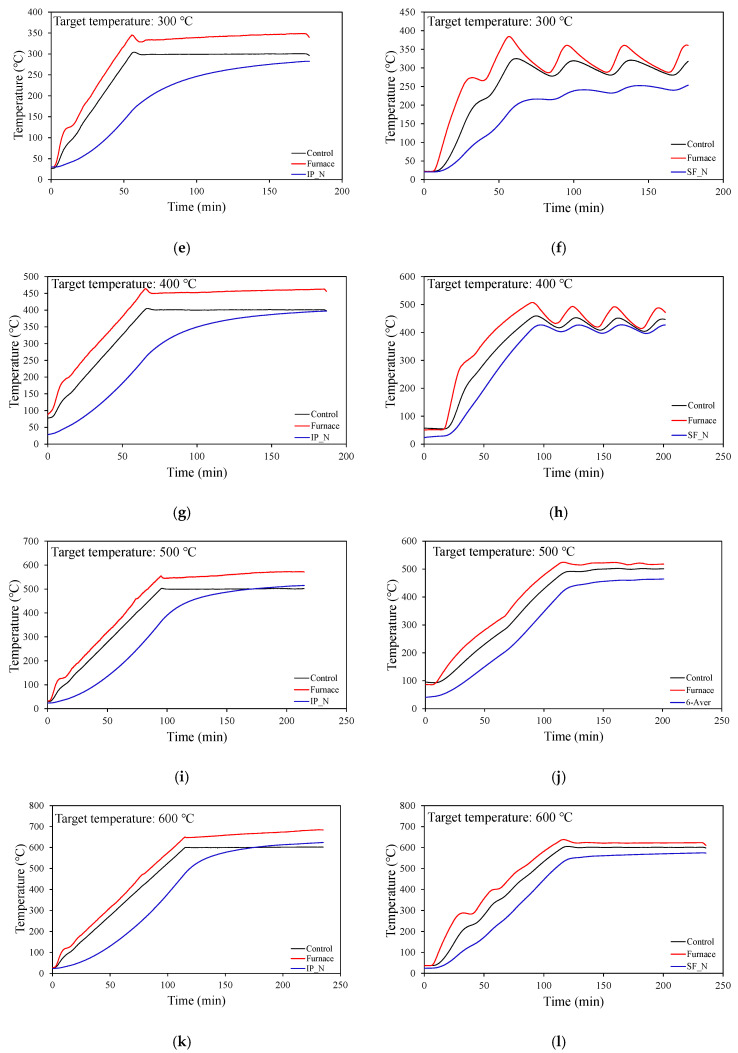

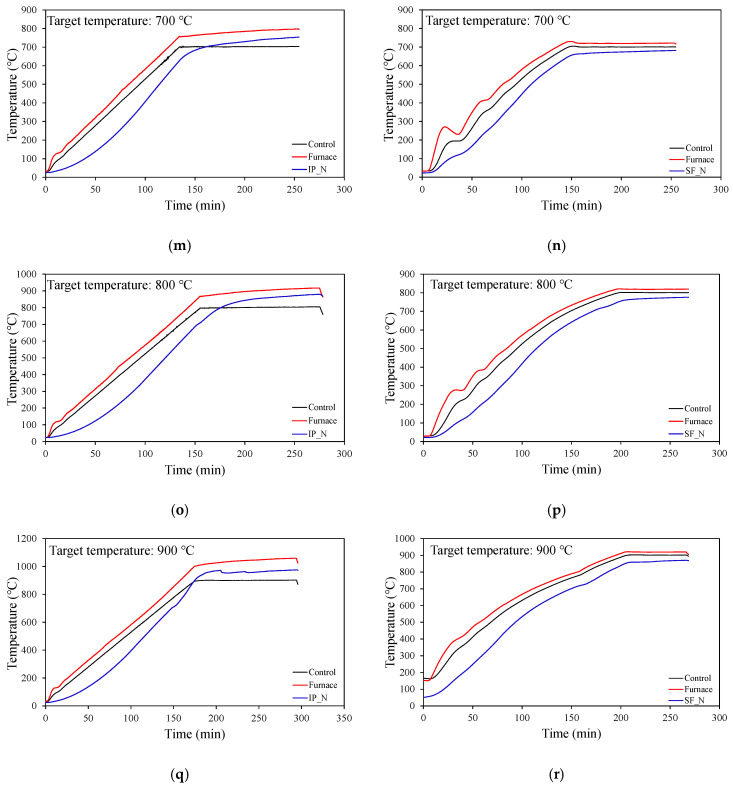
Average temperatures of furnace and non-coating specimens. (**a**) IP—Target temperature: 100 °C; (**b**) SFRM—Target temperature: 100 °C; (**c**) IP—Target temperature: 200 °C; (**d**) SFRM—Target temperature: 200 °C; (**e**) IP—Target temperature: 300 °C; (**f**) SFRM—Target temperature: 300 °C; (**g**) IP—Target temperature: 400 °C; (**h**) SFRM—Target temperature: 400 °C; (**i**) IP—Target temperature: 500 °C; (**j**) SFRM—Target temperature: 500 °C; (**k**) IP—Target temperature: 600 °C; (**l**) SFRM—Target temperature: 600 °C; (**m**) IP—Target temperature: 700 °C; (**n**) SFRM—Target temperature: 700 °C; (**o**) IP—Target temperature: 800 °C; (**p**) SFRM—Target temperature: 800 °C; (**q**) IP—Target temperature: 900 °C; (**r**) SFRM—Target temperature: 900 °C.

After the temperature of the furnace reached the target temperature, a 2 h temperature holding period was set to allow all sections of the uncoated specimen to reach the same steady state as the target temperature. After the holding period, the furnace was shut off to cool down. Figure 3 shows the temperature of the furnace and specimens measured during every experiment. As mentioned above, the furnace temperature reached the target temperature relatively quickly in the 100 °C and 200 °C experiments on the intumescent paint panel due to applying different heating rates. Also, in the case of the furnace in the experiment on the spray-on fireproofing specimens, its temperature repeatedly rose and fell at relatively low temperatures due to the poor output control of the electric heater. However, this phenomenon occurred close to the target temperature, and the temperature measured inside the furnace and the temperature of the uncoated specimens were relatively higher in the furnace used in the intumescent paint experiment. As mentioned above, this is understood to be caused by using different furnaces in the two experiments due to reasons such as damage, and the output of each furnace was also different.

**Figure 4:**
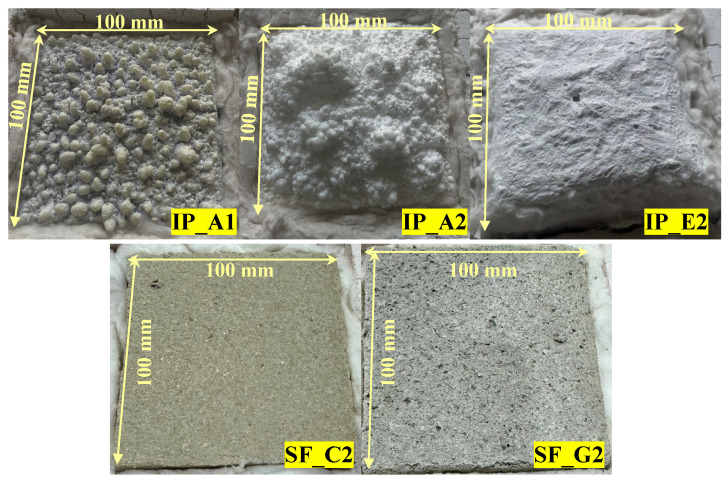
Specimen after heating and cooling (target temperature: 900 °C).

Figure 4 shows images of the specimens after the heating experiment was conducted. No external changes were observed in the spray-on fireproofing specimens. However, the intumescent paint specimens expanded according to their insulation mechanism in the event of a fire from the target exposure temperature of 200 °C. In particular, the specimens expanded into different shapes depending on the type of paint. The epoxy-type specimen expanded evenly over the entire coated surface, but the acrylic-type showed a relatively high expansion rate in unspecified areas. These expansion shapes may cause differences in insulation performance.

## 3. Results and Discussion

**Figure 5:**
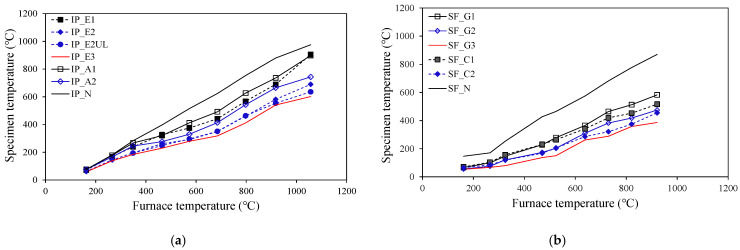
Temperatures of the specimen. (**a**) Intumescent paint; (**b**) spray-on fireproofing.

Figure 5 shows the temperature of each specimen measured during the experiment. As mentioned in Figure 4, the difference in expansion geometry caused by the type of intumescent paint resulted in temperature differences in the specimens with the same fireproof reinforcement (IP_A1 and IP_E1, IP_A2 and IP_E2). In the spray-on fireproofing test, the cement-type specimens showed relatively better insulation performance than gypsum-type materials. The insulation performance of spray-on fireproofing was also relatively better than the IP_N and SF_N specimens, depending on the temperature of each specimen. In this regard, if further research is conducted on material properties, including thermal conductivity according to the exposure temperature of each material, more diverse information in selecting fire protection materials can be used.

**Figure 6:**
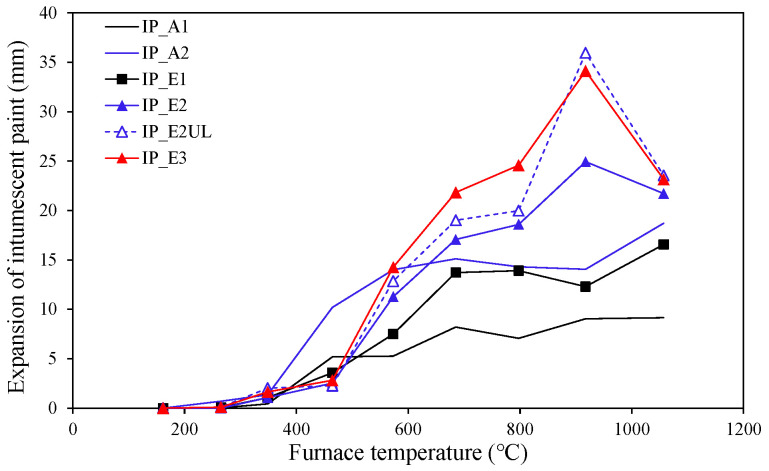
Expansion of intumescent paint [26].

Figure 6 shows the maximum expansion of intumescent paint according to the furnace temperature, where specimens with relatively high fire resistance performance have large expansion values. The maximum expansion of the acrylic-type specimens was measured where the coating surface expanded evenly, but was not measured in the uneven areas shown in Figure 4. Except for IP_E1, the expansion value of the epoxy-type specimens started to decrease when the exposure temperature exceeded 1000 °C. This phenomenon was caused by the effects of combustion or phase change of paint components in a certain temperature range. However, there is no research available on this matter or information provided by the manufacturer, so further research will be necessary. In general, manufacturers only conduct performance tests in accordance with relevant regulations and test methods to satisfy product performance standards and do not consider experimental studies that set target exposure temperatures and temperature-holding interval settings, such as those conducted in this study [26]. Building fires can have various maximum temperature ranges and fire durations depending on the circumstances. Therefore, additional experimental and theoretical studies considering various fire exposure conditions other than the standard fire curve are needed to provide more material data.

**Figure 7:**
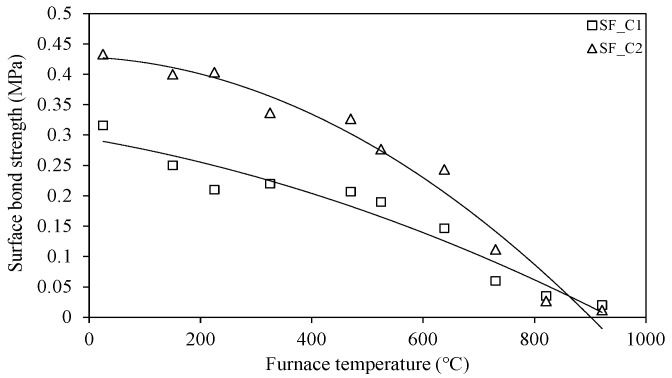
Surface bond strength of cement-type spray-on fireproofing.

**Figure 8:**
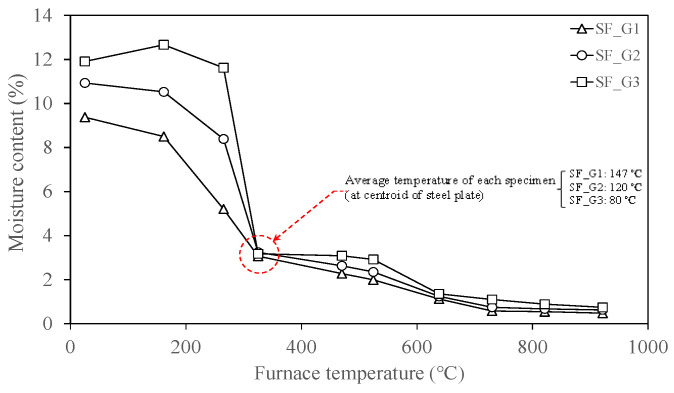
Moisture contents of gypsum-type spray-on fireproofing.

As shown in Table 1, gypsum- and cement-type SFRMs were used in this study. Gypsum-type SFRMs have low strength and stiffness and exhibit no visual difference before and after fire damage. Cement-type SFRMs also do not show visual differences before and after fire damage, but their strength and stiffness are relatively greater than gypsum-type SFRMs. Cho et al. [27] measured the surface bond strength of SFRMs according to the time they were exposed to standard fire curve conditions. The results showed that the surface bond strength decreased as the fire intensity and exposure time increased. In the case of gypsum-type SFRMs, their measured bond strength was insignificant due to their relatively low strength and stiffness, as mentioned earlier. In this study, the surface bond strength of cement-type SFRMs was measured based on the study by Cho et al. [27], as shown in Figure 7. The surface bond strength was measured using the Proceq DY-206 pull-off adhesion tester after attaching a 50 mm diameter circular disk to the surface of the specimen. Similar to previous studies, the bond strength decreased as the exposure temperature increased. As mentioned above, gypsum-type SFRMs have low strength and stiffness, so it was impossible to measure the bond strength of the surface. Therefore, a separate material characterization analysis was conducted. Although there were no relevant cases found in the literature, the moisture content of the material was measured based on the wet construction method. Figure 8 shows the results of measuring the moisture content of gypsum-type SFRMs. The moisture content was measured by taking samples between the surfaces of the steel plates at a depth in the center, rather than on the surface where the moisture content is relatively low due to direct exposure to high temperatures. The results showed the difference in moisture content varied significantly based on the target temperature of 300 °C. This means that at the target temperature of 300 °C, the temperature of the center of the steel plate of all specimens exceeded or was near 100 °C (the boiling point of water). In addition, the temperature of the sample location where the moisture content was measured exceeded 100 °C, resulting in a rapid increase in moisture evaporation. Also, the moisture content of the room temperature specimen (SF_G3) was lower than that of the target temperature 100 °C specimen.

**Figure 9:**
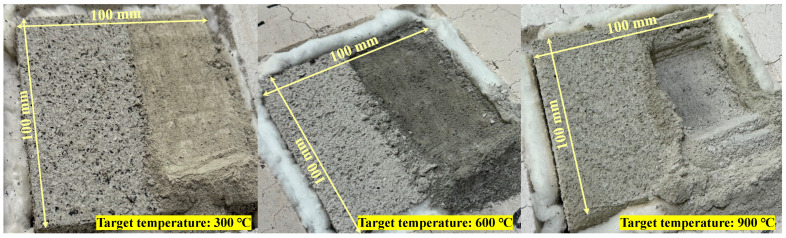
Discoloration of gypsum-type spray-on fireproofing.

**Figure 10:**
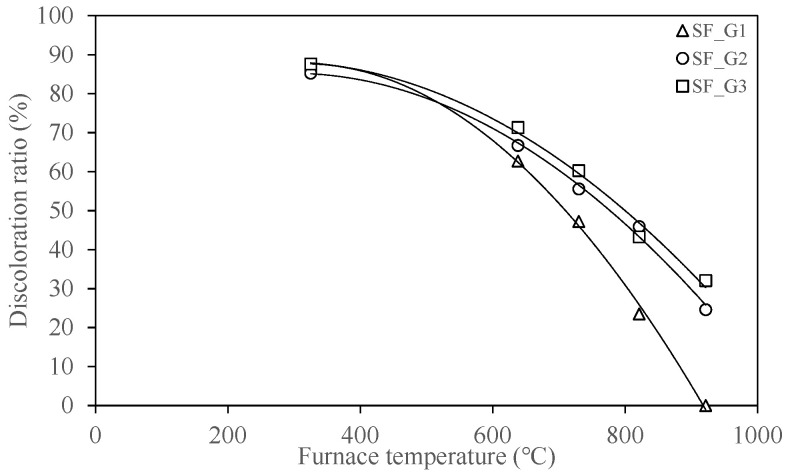
Discoloration ratio of gypsum-type spray-on fireproofing.

This phenomenon occurred because the room temperature specimen’s SFRM and steel plate were separated after preparing the specimen, which was caused by a large amount of internal moisture evaporation due to drying of the surface of the room temperature specimen. The surface of this specimen also had a relatively large area exposed to the outside air. As shown in Figure 9, during the sampling process for measuring moisture content, it was observed that the internal and external colors of the samples were different. In the middle of the sampling process, this discoloration trend started at a certain depth depending on the exposure temperature, and Figure 10 shows the measured discoloration ratios.

The discoloration ratio in Figure 10 is the ratio of the discolored thickness to the thickness of the gypsum-type SFRM, which was determined by removing the sample from the surface towards where the steel plate was attached while taking samples for measuring moisture content. The discoloration of the materials shown in Figure 9 and Figure 10 is also believed to be related to the moisture content shown in Figure 8. Observations during the sampling process revealed that the more the samples were exposed to relatively high temperatures, the lighter the discolored color became. In addition, the specimens with a target temperature of 300 °C had a fiber texture similar to that of the room temperature specimen, while the texture of the 600 °C specimens was closer to powder. These changes in color and texture were caused by the evaporation of moisture, and detailed characterization, such as dehydration and decomposition of components due to the exposure temperature of concrete, should be confirmed through further research.

## 4. Conclusions

This study analyzed the properties of fire protection materials applied to steel frame structures according to exposed temperatures as part of a research project to develop diagnostic evaluation procedures for steel frame structures damaged by fire. Various behavioral evaluations and analyses were conducted on heated and cooled specimens of spray-on fireproofing and intumescent paints commonly used in many countries, including Korea. The main findings are as follows:

1. The epoxy-type intumescent paint expanded evenly across all coated surfaces, whereas the acrylic-type paint expanded unevenly in some areas. This phenomenon confirmed that the insulation performance of intumescent paints varies by approximately 6% to 25% depending on the type of material used.
2. The cement-type SFRM exhibited a decrease in surface bond strength of about 8% to 92% compared to room temperature, depending on the exposure temperature. This reduction in bond strength can lead to cross-sectional losses, such as concrete spalling, depending on the moisture content and end constraint, resulting in a significant decrease in insulation performance.
3. This study is the first to present moisture content data for gypsum-type SFRM based on exposure temperature. It was found that the depth of discoloration varied according to exposure temperature during the sampling process for measuring moisture content.
4. The SFRMs were relatively superior (approximately 17% to 25%) in insulation performance, which is the fundamental reason for using spray-on fireproofing and intumescent paints. Although intumescent paints have relatively high construction costs, they have been widely used in recent years due to their aesthetic excellence and thermal insulation performance, even though construction delays may occur depending on their required thickness. On the other hand, SFRMs are more efficient and can save construction time and reduce costs. So, the pros and cons should be compared when selecting the most suitable fire protection material to apply.
5. The material characterization data of spray-on fireproofing and intumescent paints according to exposed temperatures presented in this study will be used for fire diagnosis procedures for steel frame structures. The most crucial factor in the fire diagnosis process of a structure is the heating temperature applied to the structural members during a fire [28], which can be estimated using the data derived from this study.

## Figures and Tables

**Table 1:** Variables for the material heating experiment.

Experimental Series (Paint and Sprayed)		Thickness of Fireproof Material—‘Data ID’ (Fire Resistance Certification Time, Hour)	Steel Plate		Target Temperature °C
			Plane (mm)	Thickness (mm)	
Intumescent Paint (IP)	Epoxy type	2.65 mm—‘IP_E1’ (1 H)	100 × 100	20	25 (ambient) 100 200 300 400 500 600 700 800 900
		8.90 mm—‘IP_E2’ (2 H)			
		9.22 mm—‘IP_E2UL’ (2 H in UL)			
		11.20 mm—‘IP_E3’ (3 H)			
	Acrylic type	0.75 mm—‘IP_A1’ (1 H)			
		2.60 mm—‘IP_A2’ (2 H)			
	Non-cover	0 mm—‘IP_N’			
Spray-on Fireproofing (SFRM)	Gypsum type	10 mm—‘SF_G1’ (1 H)			
		20 mm—‘SF_G2’ (2 H)			
		30 mm—‘SF_G3’ (3 H)			
	Cement type	21 mm—‘SF_C1’ (1 H)			
		32 mm—‘SF_C2’ (2 H)			
	Non-cover	0 mm—‘SF_N’			

## Data Availability

The original contributions presented in the study are included in the article, further inquiries can be directed to the corresponding author.
